# Recent developments of novel matrices and on-tissue chemical derivatization reagents for MALDI-MSI

**DOI:** 10.1007/s00216-020-03023-7

**Published:** 2020-11-19

**Authors:** Qiuqin Zhou, Annabelle Fülöp, Carsten Hopf

**Affiliations:** Center for Mass Spectrometry and Optical Spectroscopy (CeMOS), Mannheim University of Applied Sciences, Paul-Wittsack-Str. 10, 68163 Mannheim, Germany

**Keywords:** MALDI-MSI, MALDI matrix, MALDI imaging, On-tissue chemical derivatization, Tissue preparation

## Abstract

Matrix-assisted laser desorption/ionization mass spectrometry imaging (MALDI-MSI) is a fast-growing technique for visualization of the spatial distribution of the small molecular and macromolecular biomolecules in tissue sections. Challenges in MALDI-MSI, such as poor sensitivity for some classes of molecules or limited specificity, for instance resulting from the presence of isobaric molecules or limited resolving power of the instrument, have encouraged the MSI scientific community to improve MALDI-MSI sample preparation workflows with innovations in chemistry. Recent developments of novel small organic MALDI matrices play a part in the improvement of image quality and the expansion of the application areas of MALDI-MSI. This includes rationally designed/synthesized as well as commercially available small organic molecules whose superior matrix properties in comparison with common matrices have only recently been discovered. Furthermore, on-tissue chemical derivatization (OTCD) processes get more focused attention, because of their advantages for localization of poorly ionizable metabolites and their‚ in several cases‚ more specific imaging of metabolites in tissue sections. This review will provide an overview about the latest developments of novel small organic matrices and on-tissue chemical derivatization reagents for MALDI-MSI.

**Figure:**
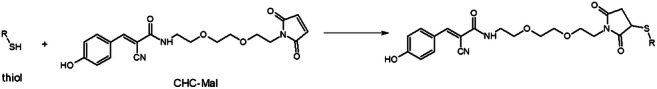
Graphical abstract

Graphical abstract

## Introduction

**Fig. 1:**
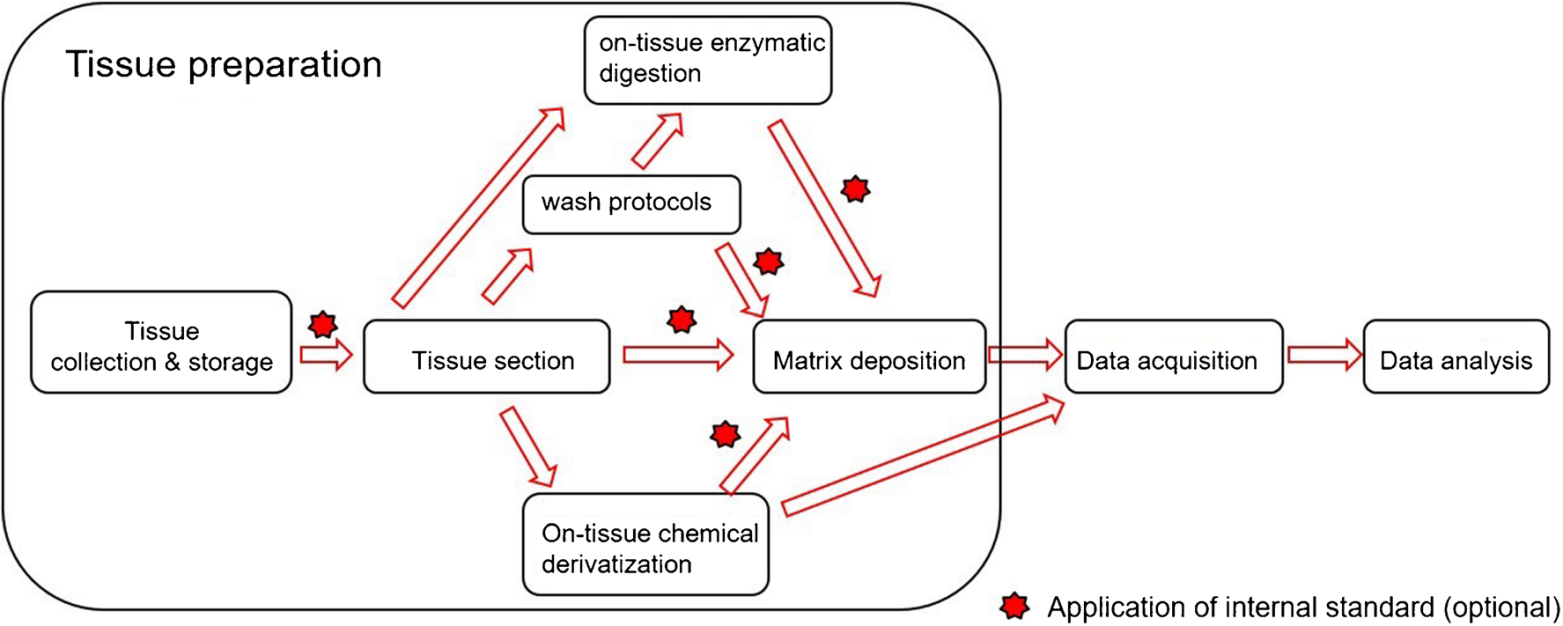
Schema of experimental workflow for MALDI-MSI with highlighted important steps for tissue preparation

Matrix-assisted laser desorption/ionization (MALDI) time-of-flight (TOF) mass spectrometry imaging (MSI) can provide unparalleled insight into the spatial distribution of proteins [1], peptides [2], small molecules [3], lipids [4], glycans [5], and drugs [6] in tissue sections. The fast-evolving MALDI-MSI technique has been successfully applied in basic research, in pharmaceutical research [6], plant biology [7], food analysis [8], microbiology [9], and in clinical biomarker discovery [10]. In short, typical MALDI-MSI workflows contain the following three steps: tissue preparation, data acquisition, and data analysis. The “4S-criteria” for a desirable MSI experiment [6], namely **s**peed, **s**pecificity, **s**patial resolution, and **s**ensitivity, often cannot be achieved together, and compromises are required for MALDI-MSI methods. Although developments of mass spectrometer hardware have a great impact on the performance of MALDI-MSI, proper tissue preparation [11–14] is key for high-quality data acquisition. During tissue preparation, it is essential to preserve the fidelity of analytes and prevent their spatial dislocation in tissue. Additionally, time consumption for tissue preparation should stay within an acceptable range, in most cases several hours. Important steps for tissue preparation are illustrated in Fig. 1.

Different workflows from tissue collection to data acquisition can be followed for tissue preparation. Depending on tissue type, targeted analyte species and on experimental goals, washes, on-tissue enzymatic digestion (OTED), on-tissue chemical derivatization (OTCD), and application of internal standard are options in addition to the main experimental workflow (Fig. 1) [12, 15, 16]. Initially, procedures for collecting, storing, embedding, and sectioning of tissue samples have great influence on the resulting quality and need to be standardized for the same types of tissue. Later, wash protocols can be applied to remove interfering endogenous compounds, which compete with analytes during ionization and cause ion suppression effects. For example, wash protocols are indispensable for imaging of peptides and proteins in tissue sections to get rid of physiological salts and abundant lipids. Furthermore, wash protocols can also be used to enhance the subsequent ionization or extraction of the analytes into the matrix layer [17–19]. OTED belongs to often-used strategies for identification and visualization of proteins [2] and glycans [5]. Finally, before starting data acquisition, the matrix needs to be applied homogenously except that reagents used in the optional OTCD step can comply the requirement and replace of the additional matrix application. Choosing a suitable matrix and a suitable deposition method can avoid missing analytes or other time-consuming tissue preparation steps [13, 20]. Aiming at improvement of detection specificity and enhancement of detection sensitivity in some approaches, OTCD has been specially developed for small molecule analytes [21]. To compensate ion suppression effects in heterogeneous tissue sections, internal standard can be used for normalization and relative quantification, yielding superiorly comparable results from pixel to pixel and tissue to tissue [6, 22, 23]. This work only selected the latest developments of novel matrices and on-tissue chemical derivatization reagents for MALDI-MSI.

## Novel matrices for MALDI-MSI

Ideal matrices for MALDI-MSI need several additional features in comparison with matrices for MALDI-MS used in non-imaging fields. There are some fundamental properties for substances that can be used as MALDI matrix for all applications: (i) MALDI matrices display strong and efficient absorption in the region of commonly used UV or IR laser wavelengths; (ii) minimal matrix background signals and adducts with target analytes (“chemical noise”) should result from matrices, especially in the low mass range (typically *m/z* < 500), thus enabling visualization of the spatial distribution of small molecules; (iii) the ability to interact with analytes is required for matrices to form co-crystals; (iv) matrices must be able to effectively ionize the analytes, resulting in protonated ions in positive ion mode or deprotonated ions in negative ion mode. In addition to these general requirements for MALDI matrices, a matrix suitable for tissue imaging applications should fulfill the criteria: (A) It should have high vacuum stability in the mass spectrometer for at least several hours in a typical imaging situation or for even longer acquisition times in serial imaging of multiple tissue sections, for example, entire clinical cohorts, with high spatial resolution. (B) The application of matrices onto tissue should be practical in a routine laboratory, i.e., reproducible and not time-consuming. Sublimation and automatic spray-coating methods are often used due to easy handling and sufficient reproducibility. (C) The generation of uniform, small co-crystals is necessary for images with reproducibility and high spatial resolution, which is also limited by minimal laser spot size and the desired sensitivity (since the ablated area, which decreases with the square of the step size, strongly correlates with sensitivity). Thus, high spatial resolution requires an optimized matrix, which can promote efficient ionization of target analytes. (D) For the untargeted discovery of biomarkers, matrices suitable for both positive and negative ion modes (also called dual-polarity) cannot only save precious tissue but also provide broader molecular coverage. The ability of ionizing more types of analytes in single ion mode (positive or negative) would be beneficial for detecting more molecular species simultaneously. (E) Owing to physiologic salts naturally present in tissue, salt tolerance allows the direct application of matrices without additional wash protocols. Finally, chemical stability, cost, toxicity, and promotion of analyte fragmentation need to be considered. In the field of MALDI-MSI, performance with desired detection sensitivity, spatial resolution, and molecular coverage is in large part dependent on matrix choices and matrix application. Because MALDI-MSI is becoming more and more popular in different disciplines and the existing matrices do not always meet all expectations stated above, many laboratories are developing novel matrices with at least some of the abovementioned requirements.

**Fig. 2:**
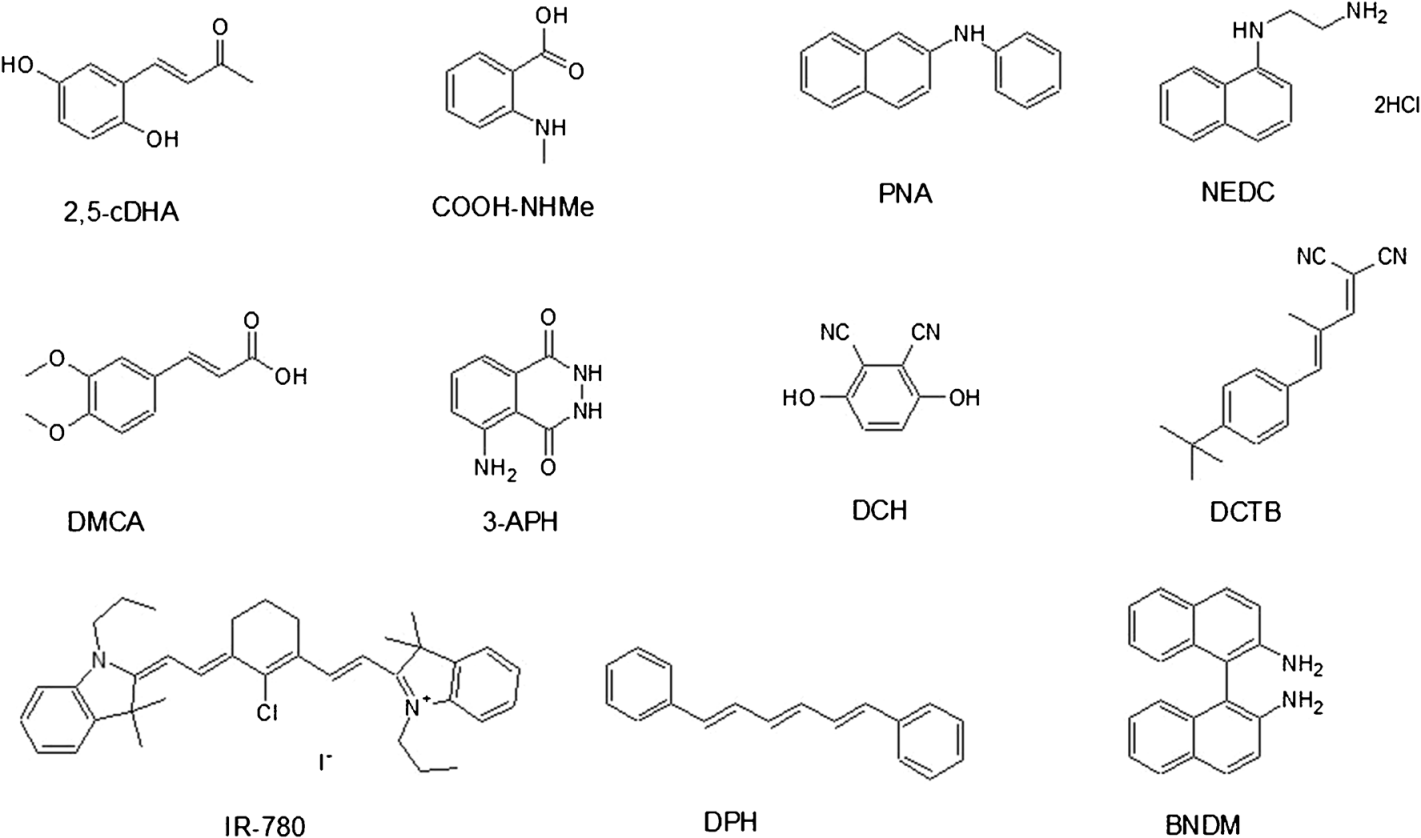
Structures of selected novel MALDI matrices for imaging

Currently, most very oft-used MALDI-MSI matrices like 2,5-DHB and CHCA were developed for specific analytes during empiric research. Different substance classes, such as small organic molecules, graphene, graphene oxide, nanoparticles, metal oxides, ionic liquids, and conjugated polymers, are presently developed, tested for their suitability as MALDI-MSI matrices, and reviewed by various papers [4, 11, 20]. Small organic molecules are the most widespread matrices used in MALDI-MSI. Since recent reviews [11, 20] have already described some novel organic matrices applied for MALDI-MSI including 1,5-diaminonapthalene [24], 4-phenyl-a-cyanocinnamic acid amide [25], alkylated 2,5-DHB [26], 1,8-di(piperidinyl)-naphthalene [27], and 4,5-(bis(dimethylamino)naphthalen-1-yl)furan-2,5-dione (4-maleicanhydrido proton sponge, MAPS) [28], this work selected only the latest efforts about novel organic matrices for MALDI-MSI from 2017. This section contains the rationally designed and synthesized as well as commercially available small organic molecules whose properties as bona fide MALDI matrices for the detection and imaging of analytes in tissue sections have recently been discovered or characterized (Fig. 2 and Table 1).

**Table 1:** Rationally designed novel matrices and newly discovered novel matrices for MALDI-MSI

Matrix abbr.	Matrix	Target analyte classes	Ion modes	Properties	Deposition	Ref.
• Rationally designed small organic substances as novel matrices						
2,5-cDHA	(E)-4-(2,5-dihydroxyphenyl)but-3-en-2-one	Protein, lipid	+, − *	More vacuum-stable, small crystals for higher spatial resolution, improved sensitivity, dual-polarity for lipids	Two-step spraying	[29]
COOH-NHMe	2-(Methylamino)benzoic acid	Lipid, protein	+, −	Dual-polarity, detection of more lipid and protein species	Sublimation	[30]
• Newly discovered small organic substances as novel matrices						
PNA	N-Phenyl-2-naphthylamine	FA, AA, antioxidant, lipid	–	Strong UV-absorption, low matrix background signals, salt tolerance capacity	Spraying	[31]
NEDC	N-(1-Naphthyl)ethylenediamine dihydrochloride	Glucose, Na^+^, K^+^, AA, nucleotide, antioxidant, glycerophospholipid	–	Salt tolerance capacity, low matrix background signals, improved sensitivity for selected analyte classes	Spraying	[32–36]
3-APH	3-Aminophthalhydrazide	Nucleotide, FA, lipid	+, −	Dual-polarity, improved sensitivity, broad molecular coverage, low matrix background signals, vacuum stability	Spraying	[37]
IR-780		(Poly)phosphoinositide, cardiolipin, ganglioside	–	Proton affinity, vacuum stability, homogenous crystals, salt tolerance capacity	Spraying	[28]
DPH	1,6-Diphenyl-1,3,5-hexatriene	Lipid, FA with polyene structure	–	Vacuum stability, high spatial resolution	Sublimation	[38]
BNDM	1,1′-Binaphthyl-2,2′-diamine	AA, organic acid, nucleoside, nucleotide, nitrogenous base, cholesterol, peptide, FA, choline, carnitine, polyamine, creatine, lipid	+, −	Low matrix background signals, improved sensitivity, dual-polarity, broad molecular coverage	Spraying	[39, 40]
DCH	2,3-Dicyanohydroquinone	Lipid	+	High spatial resolution, vacuum stability, chemical stability, sensitivity	Spraying	[41]
DCTB	(2-[(2E)-3-(4-Tert-butylphenyl)-2-methylprop-2-enylidene]malononitrile)	Central nervous system drug	+	Improved sensitivity, low signal suppression	Spraying	[42]
DMCA	3,4-Dimethoxycinnamic acid	Small molecule	+	Low matrix background signals, sensitivity, broad molecular coverage	Spraying	[43]

*Positive ion mode for protein and dual-polarity for lipid. *AA* amino acid, *FA* fatty acid

### Rationally designed and synthesized novel matrices

(E)-4-(2,5-Dihydroxyphenyl)but-3-en-2-one (2,5-cDHA) was designed and synthesized by Yang and coworkers [29] among other derivatives, using Wittig reaction of 2′,5′-dihydroxybenzaldehyde with (acetylmethylene)-triphenylphosphorane. 2,5-cDHA was applied to tissue by a two-step spraying approach, producing small crystals (1–2 μm). Unlike the parent matrix 2,5-DHA, the 2,5-cDHA proved to be vacuum-stable for more than 24 h in the Bruker Rapiflex mass spectrometer, whose source has a high vacuum (4.4 * 10^−7^ mbar) and heated (around 35 °C) environment. As a result, 2,5-cDHA was used for the visualization of proteins in mouse brain at high spatial resolution (< 10 μm) and for many hours without concern of matrix loss in the mass spectrometer. Additionally, 2,5-cDHA was also used for imaging of lipids in both positive and negative ion modes.

Huang and coworkers [30] rationally designed and synthesized a series of novel matrix candidates, including 2-(methylamino)benzoic acid (COOH-NHMe), candidate as a MALDI matrix for imaging. The matrix COOH-NHMe was synthesized by adding a methyl group to anthranilic acid using CH_3_I in basic conditions. The new methylated anthranilic acid derivative features both basic and acidic functional groups and thus showed the ability to be used in both positive and negative ion modes for the ionization and detection of lipids and proteins in mouse brain tissue. In comparison with commercially available matrices, COOH-NHMe was able to visualize more lipid and protein species.

### Newly discovered novel matrices

N-Phenyl-2-naphthylamine (PNA) is usually used as an antioxidant for natural and synthetic rubber. Liu and coworkers [31] developed PNA as a matrix for imaging of small-molecule metabolites, including free fatty acids, amino acids, antioxidants, and phospholipids. On account of its strong UV absorption, low background interference in the *m/z* range of < 500 and considerable salt tolerance, the unique distributions and changes of 89 small-molecule metabolites were visualized in negative ion mode in brain tissue of a middle cerebral artery occlusion (MCAO) rat model of ischemic stroke.

N-(1-Naphthyl)ethylenediamine dihydrochloride (NEDC) has been known as a commercially available coupling agent for spectrophotometric analysis. Since Chen and coworkers [33] discovered NEDC as a high-salt tolerance matrix for the analysis of glucose as [glucose + Cl^−^]^−^ in rat brain microdialysates in negative ion mode, several contributions of NEDC could be found in the MS imaging field. Hou and coworkers [34] applied NEDC to mouse brain sections for visualization of sodium and potassium distribution. Here, sodium and potassium were detected as chloride ion adducts. Wang and coworkers [36] were able to visualize distributions of glycerophospholipids and small-molecule metabolites below *m/z* 400, including glucose in various mouse organs. Li and coworkers [35] successfully used NEDC for imaging of the change of lipid metabolism and the levels of amino acids, nucleotides, and antioxidants in brain tissue of an orthotopic glioma xenograft model. Barré and coworkers [36] compared NEDC with 9-AA for imaging of small metabolites in diffuse large B cell lymphoma xenograft mouse model. With a higher signal-to-noise ratio for metabolites and less matrix background, NEDC appears to be a better matrix for adenosine monophosphate (AMP), adenosine diphosphate (ADP), and adenosine triphosphate (ATP) than 9-AA.

3-Aminophthalhydrazide (3-APH, also known as luminol) is a commercially available and very effective chemiluminescent substrate. 3-APH was evaluated by Li and coworkers [37] as a dual-polarity matrix with superior performance than common matrices such as 2,5-DHB, CHCA, and 9-AA. 3-APH was able to detect and give a spatial distribution of 159 and 207 metabolites in the mouse brain in the positive and negative ion modes, respectively. Among the detected metabolites were nucleotides, fatty acids, glycerolipids, glycerophospholipids, sphingolipids, and saccharolipids. Thus, 3-APH was demonstrated to have some improved features including high sensitivity, broad molecular coverage, low background noise, and high vacuum stability.

IR-780 is a commercially available near-infrared fluorescent dye. Li and coworkers [28] successfully screened and optimized the application of IR-780 as a matrix for visualization of various high-molecule lipids, including (poly-)phosphoinositides, cardiolipins, and gangliosides in acute traumatic brain injury tissues in negative ion mode. IR-780 was proved to possess various good properties including strong UV absorption, high proton affinity, good salt tolerance ability, homogeneous co-crystallization, and high vacuum stability. Thus, it was suggested as an almost ideal matrix for imaging.

1,6-Diphenyl-1,3,5-hexatriene (DPH) is a commercially available fluorescent dye for the detection and localization of lipids in fluorescence imaging. Ibrahim and coworkers [38] presented DPH as a suitable and effective matrix for imaging of lipids and fatty acids in rat and mouse brain tissues in negative ion mode. It was proposed that the interaction with the acyl chain of lipids and fatty acids is accountable for the features of DPH as a matrix for lipids and fatty acids with hydrophobic polyene structure. The possibility of application via sublimation and the stability for at least 24 h under high vacuum (10–7 Torr) allowed DPH for the application for high spatial resolution imaging. Use of relative low laser energy avoided the fragmentation of lipids.

The enantiomers and derivatives of commercial available 1,1′-Binaphthyl-2,2′-diamine (BNDM) are widely used in asymmetric syntheses. Sun and coworkers [40] developed BNDM as a dual-polarity matrix with low background interference and high sensitivity. BNDM could be applied for imaging of 301 negative metabolite ions and 175 positive metabolite ions, including amino acids, organic acids, nucleosides, nucleotides, nitrogenous bases, cholesterols, peptides, fatty acids, cholines, carnitines, polyamines, creatine, and phospholipids in rat brain. Additionally, BNDM could give the spatial information of various metabolites in different mouse lung cancer tissue sections. In another work, Sun and coworkers [39] optimized BNDM for the imaging application of amino acids, phenolic acids, fatty acids, oligosaccharides, cholines, polyamines, tanshinones, and phospholipids in *Salvia miltiorrhiza* Bge.

2,3-Dicyanohydroquinone (DCH) is originally known as a fluorescent dye and was applied by Liu and coworkers [41] as a new matrix for imaging of lipids in biological tissues. In contrast to commonly used matrices, more lipids could be detected from mouse brain and germinating Chinese yew seed tissue sections in positive ion mode. Strong UV absorption, high vacuum stability, profound chemical stability, and formation of homogenous small crystals make for a good matrix with the superior performance, including high spatial resolution and reproducibility.

In 2000, Ulmer and coworkers [44] proved DCTB (2-[(2E)-3-(4-tert-butylphenyl)-2-methylprop-2-enylidene]malononitrile) as an electron transfer matrix for the analysis of labile compounds. Recently, Rzagalinski and coworkers [42] found the theoretical and experimental evidence that DCTB is a proton transfer matrix. DCTB was applied for imaging of central nervous system drugs in mouse brain tissue on positive ion mode. Despite the solubility and vacuum stability drawbacks, DCTB was applied for quantitative imaging of the adrenergic receptor agonist xylazine in mouse brain tissue with significant improvements of signal intensity over CHCA.

He and coworkers [43] investigated 3,4-dimethoxycinnamic acid (DMCA) as a promising matrix for imaging of endogenous low molecular weight metabolites (*m/z* < 500 Da) on rat liver, rat brain, and germinating Chinese yew seed tissue, with 303, 200, and 248 metabolites, respectively. Using DMCA, more low molecular weight metabolites (*m/z* < 500) could be detected and imaged than using other commonly used matrices such as 2,5-DHB, CHCA, 2-mercaptobenzothiazole (2-MBT), graphene oxide, and silver nanoparticles. Due to the broad molecular coverage and low background signals, DMCA was established as a powerful matrix with high ionization efficiency for small molecules.

## On-tissue chemical derivatization reagents

Nowadays, MALDI-MSI draws more and more attention as a label-free imaging technique that can visualize various metabolites in a single measurement. However, the MALDI-MSI technique faces some challenges such as detection sensitivity for small molecule analytes, extend of metabolic coverage, more comprehensive identification of isomers, and so on.

The detection of some metabolites in tissue sections by MALDI-MSI is sometimes hindered by their ionization efficiencies, and additionally, also by background interferences from MALDI matrix or tissue components, sampling size, ion suppression effects, and in-source fragmentation. Besides, the broad dynamic range of on-tissue metabolite concentrations and their diverse functional groups are responsible for distinct ionization efficiencies. To solve the detection problem of small metabolites in tissue sections, development of more effective matrices for MALDI with negligible matrix background and optimization of matrix deposition methods are just two strategies to improve their detection sensitivities. However, more strategies are required. On-tissue chemical derivatization (OTCD) is an alternative approach to improve ionization efficiency of targeted analytes in MALDI-MSI for the in situ detection of analytes. OTCD of small molecule analytes can increase their molecular mass, resulting in mass shifting the derivatization product ions out of the “chemical noise” range. Optimally, the derivatization products can be ionized more efficiently than analytes without derivatization, resulting in improved detection sensitivity in MALDI-MSI for the in situ detection. Finally, broader molecular coverage can be achieved. Furthermore, the specificity in MALDI-MSI can be improved by OTCD that allows specific chemical reactions for the identification of functional groups and their positions in target analytes. At best, OTCD reagents can serve as a reactive matrix, which cannot only selectively react with analytes in tissue sections but also assist in desorption and ionization in MALDI-MS.

Chemical derivatization in solution is a well-developed strategy for the detection of analytes in capillary electrophoresis [45, 46], gas chromatography [47], and high-performance liquid chromatography [45, 47] coupled with optical absorption spectrometry or electrospray ionization mass spectrometry (ESI) [48–51] for the following purposes: enabling of detection, improved structure elucidation in terms of structural isomers, simplified identification of functional groups, quantification, and improved molecular coverage.

**Fig. 3:**
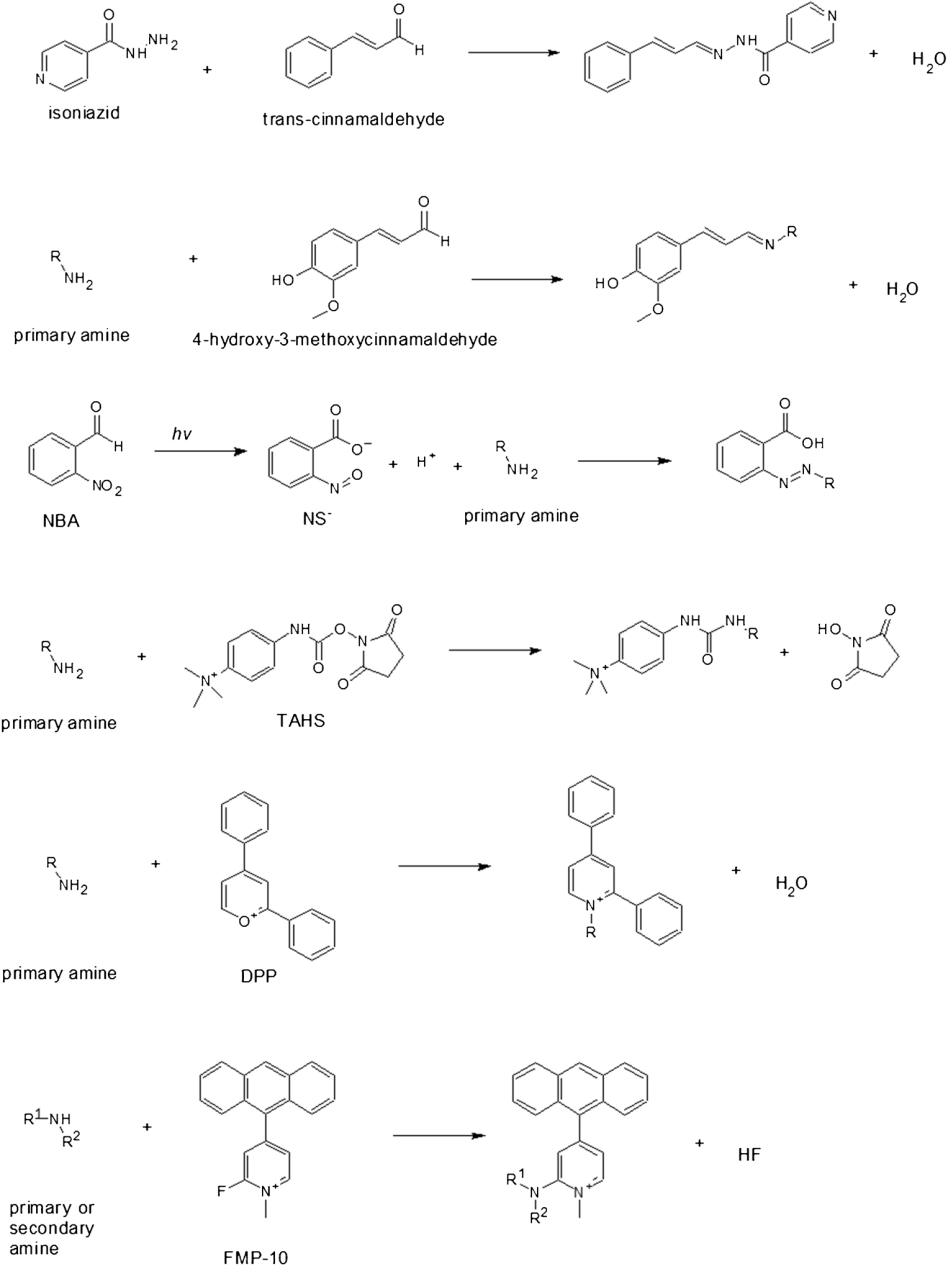
OTCD reactions targeting amines

**Fig. 4:**
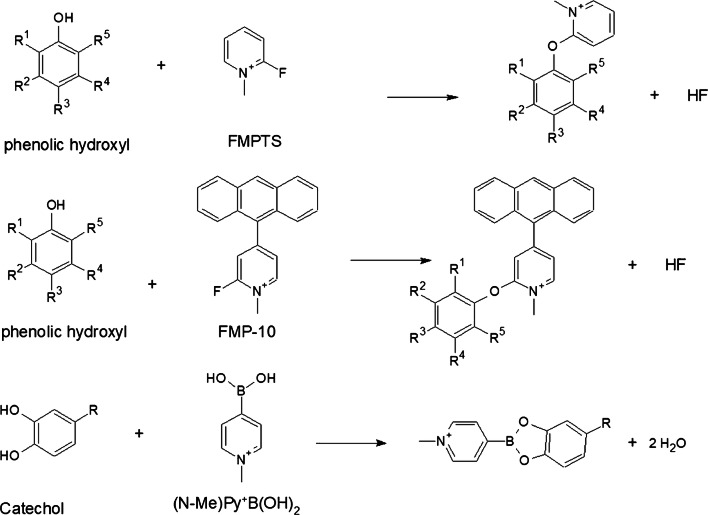
OTCD reactions targeting phenolic hydroxyl groups

**Fig. 5:**
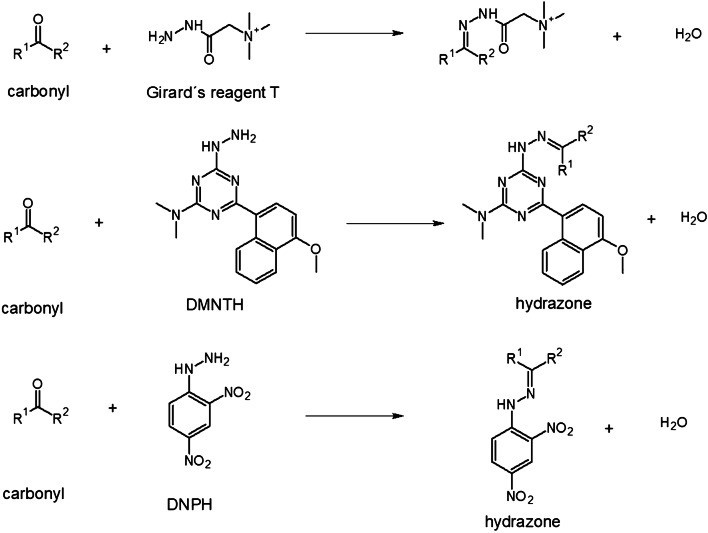
OTCD reactions targeting carbonyls

**Fig. 6:**
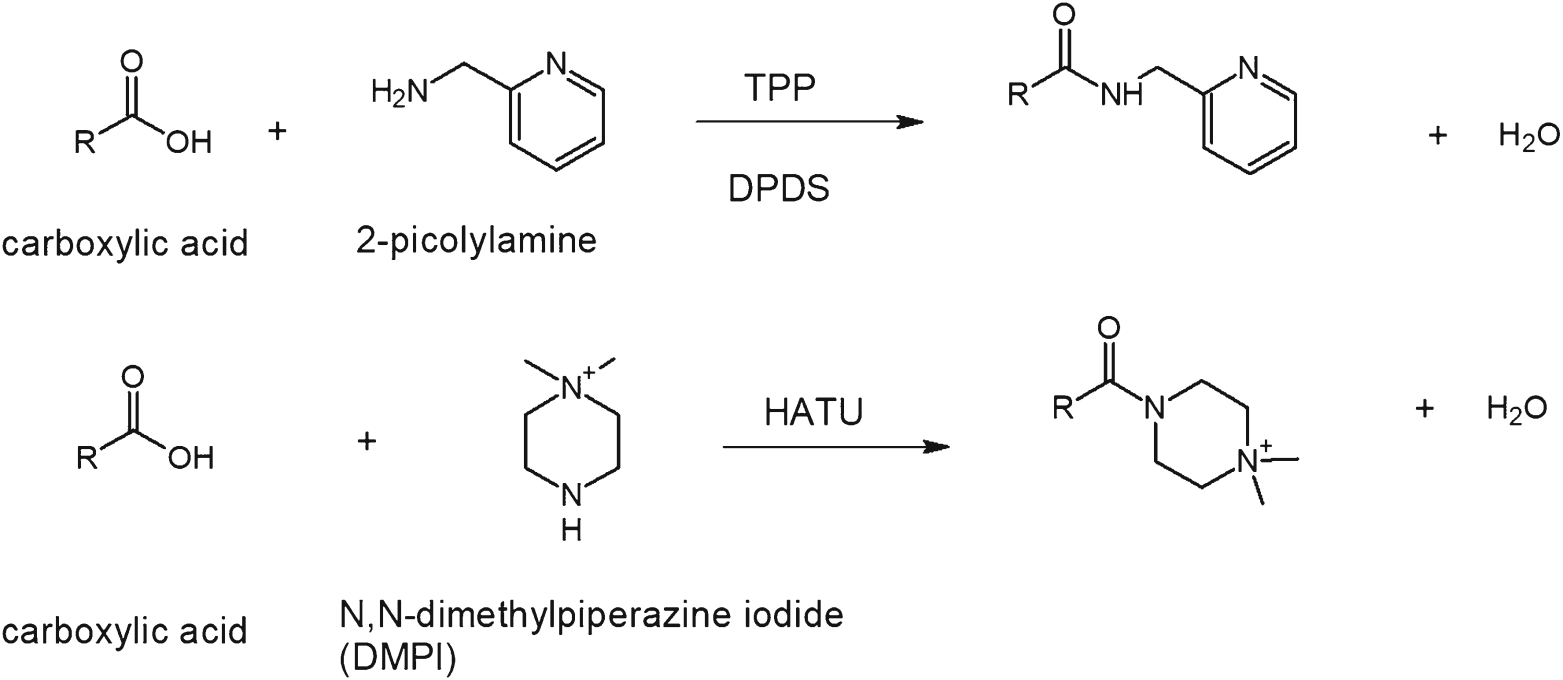
OTCD reactions targeting carboxylic acids

**Fig. 7:**
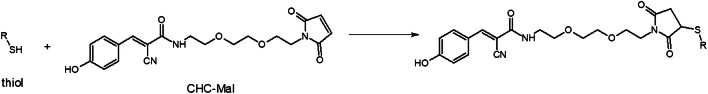
OTCD reactions targeting thiols

**Fig. 8:**
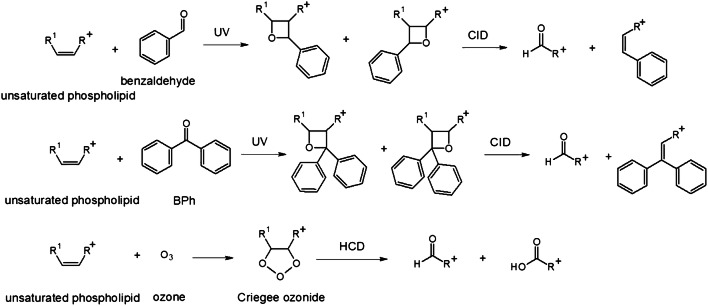
OTCD reactions targeting double bonds

In-solution chemical derivatization methods have found various applications in MALDI-MS [52]. To date, more and more chemical derivatization approaches are developed for on-tissue applications followed by MALDI-MSI measurements. In comparison to in-solution chemical derivatization, OTCD needs to overcome a couple of challenges including relatively low derivatization efficiency, interferences due to excess derivatization reagent, and delocalization due to the additional spray-coating step and incubation in the humid atmosphere. Thus, complex chemical reaction conditions often used in solution cannot easily be adapted and reconstructed for on-tissue chemical reactions. Optimally, OTCD can be performed at room temperature without the requirement of additional specific buffers and with short reaction times. Generally, OTCD employs an automatic sprayer to apply derivatization reagents directly onto tissue sections before incubation in a humid chamber at room temperature or slightly increased temperature for several hours. Several review papers [5, 53, 54] have already included the work from Holster and coworkers [55], who developed a linkage-specific two-step OTCD process for obtaining spatial distribution information of linkage-specific N-glycan isomers from FFPE tissue sections by using the MALDI-MSI technique. And although several other review papers [20, 56–58] have described new developments of mass spectrometric imaging, which described among other methods also several MALDI-MSI approaches using OTCD, we present a comprehensive overview about recent developments in OTCD of functional groups including amine, phenolic hydroxyl, carbonyl, carboxylic acid, thiol, and double bond, for applications in the MALDI-MS imaging field (Table 2 and Figs. 3, 4, 5, 6, 7, and 8).

**Table 2:** MALDI-MSI approaches with on-tissue chemical derivatization of analytes in tissue sections

Target analytes in tissue sections	OTCD reagent	Solution	Application and incubation	Tissue	Ref.
Amine as targeted functional groups					
Isoniazid	CA	50% in MeOH	High velocity spin coating, 30 min at RT	Lung tissue sections of rabbit infected with *M. tuberculosis* and dosed with isoniazid	[59]
Dopamine, norepinephrine, epinephrine, γ-aminobutyric acid	CA	23 mg/mL CA and 8.5 mg/mL *trans*-ferulic acid in MeOH	Automatic spraying	Adrenal gland tissue sections of pig, brain tissue sections of rat	[60]
Glycine, alanine, serine, proline, valine, threonine, isoleucine/leucine, aspartate, glutamine, lysine, glutamate, tryptophan, dopamine, γ-aminobutyric acid, taurine, 3-methoxytyramine, serotonin, L-dihydroxyphenylalanine	CA	4 mg/mL in 50% MeOH	Automatic spraying, overnight at RT	Brain tissue sections from female C57BL/6J mice	[61]
Glycine, alanine, aminobutenoic acid, serine, γ-aminobutyric acid and more other metabolites	CA	20 mg/mL in MeOH	Electrospraying	Leaf and root tissue sections of two different maize genotypes (B73 and Mo17)	[62]
67 small-molecule metabolites including amino acids, neurotransmitters, dipeptides and others	CA	5 mg/mL in 50% MeOH	Electrospraying, overnight at 37 °C	Brain tissue sections of rats	[63]
Neuropeptides	NBA + hv	5 mg/mL in ACN/EtOH/FA/H_2_O	Automatic spraying, nanosecond with *hv*	Brain tissue sections from mouse brain	[64]
Amino acids excluding lysine, serine, histidine, threonine, aspartate and arginine	TAHS	5 mg/mL in ACN	Airbrush, overnight at 55 °C	Liver tissue sections from xenograft mouse models of human colon cancer	[65]
Glycine, alanine, serine, proline, valine, threonine, isoleucine/leucine, aspartate, glutamine, lysine, glutamate, tryptophan, dopamine, γ-aminobutyric acid, taurine, 3-methoxytyramine, serotonin, L-dihydroxyphenylalanine	TAHS	5 mg/mL in 50% ACN	Automatic spraying, overnight at RT	Brain tissue sections from female C57BL/6 J mice	[61]
Phenylalanine, tyrosine	TAHS	5 mg/mL in ACN	Automatic spraying, 24 h at 55 °C	Liver tissue sections from a H460 human NSCLC xenograft mouse model	[66]
Noradrenaline	TAHS	5 mg/mL in ACN	Airbrush, 15 min at 55 °C	Adrenal gland tissue sections from tumor patients	[67]
Glutamine	TAHS	5 mg/mL in ACN	Automatic spraying, 24 h at 55 °C	Tumor and benign tissue sections from tumor patients	[68]
Dopamine, tyrosine, tryptamine, tyramine, phenethylamine, 3-methoxytyramine, serotonin, γ-aminobutyric acid, glutamate	DPP-TFB**, TMP-TFB	0.09 mg/mL in 50% MeOH with 0.06% TEA	Automatic spraying, 30 min at RT	Brain tissue sections from treated and control male Sprague-Dawley rats and C57BL/6J male mice, brain tissue sections from primate	[69]
Dopamine and amphetamine, β-N-methylamino-L-alanine	DPP*, TMP, PBDPP*	1.11 mg/mL in 75% MeOH with 0.05% TEA, 1 mg/mL in 83% MeOH with 0.05% TEA, 0.2 mg/mL in 80% MeOH with 0.07% TEA	Automatic spraying, 15 min at RT	Brain tissue sections from the treated and untreated C57BL/6J male mice, male Wistar rat pups or adult Sprague-Dawley rats	[70]
Glycine, alanine, serine, proline, valine, threonine, isoleucine/leucine, aspartate, glutamine, lysine, glutamate, tryptophan, dopamine, γ-aminobutyric acid, taurine, 3-methoxytyramine, serotonin, L-dihydroxyphenylalanine	DPP-TFB	5 mg/mL in MeOH	Automatic spraying, overnight at RT	Brain tissue sections from female C57BL/6J mice	[61]
Glutamate, γ-aminobutyric acid	DPP-TFB	1.33 mg/mL in MeOH	Manual spraying, direct spraying of matrix	Brain tissue sections from SCR-KO and WT mice	[71]
Dopamine, serotonin, norepinephrine	DPP-TFB	1.3 mg/mL in MeOH	Airbrush, direct spraying of matrix	Whole brain of the C57BL/6J mouse	[72]
Dopamine, 3-methoxytyramine	DPP-TFB	1.3 mg/mL in MeOH	Airbrush, direct spraying of matrix	Brain tissue sections from male C57BL/6J mice and WT mice	[73]
Dopamine, glycine, alanine, γ-aminobutyric acid, proline, valine, threonine, taurine, leucine, aspartate, tyramine, glutamine, lysine, glutamate, tryptamine, 3-methoxytyramine, tyrosine, L-dihydroxyphenylalanine	DPP-TFB	5 mg/mL in MeOH	Automatic spraying, overnight	Brain tissue sections from glioblastoma multiforme mice and WT mice	[74]
Dopamine, serotonin, γ-aminobutyric acid, histamine, threonine, phenethylamine, methylhistamine, agmatine, adenine, tyramine, lysine, tryptamine, L-dihydroxyphenylalanine	DPP-TFB	1.33 mg/mL in 75% MeOH with 0.05% TEA	Automatic spraying, 24 h at RT	Brain tissue sections from rock crabs *Cancer irroratus*	[75]
Dopaminergic and serotonergic neurotransmitters and their associated metabolites containing primary and secondary amine groups	FMP-8*, FMP-9*, FMP-10*	4.4 mM in 70% ACN	Automatic spraying, no incubation	Brain tissue sections from treated and untreated rat and primate models of Parkinsonism, brain tissue sections from a patient with Parkinson’s disease	[76]
Dopamine, γ-aminobutyric acid	FMP-10*	1.8 mg/mL in 70% ACN	Automatic spraying, no incubation	Brain tissue sections from GPR37 KO mice and WT mice	[77]
Phenolic hydroxyl as targeted functional groups					
Cannabinoids and their metabolites	FMPTS	10 mg/mL in ACN	Airbrush	Human hair	[78]
Catecholamines (dopamine, epinephrine, norepinephrine)	(N-Me)Py^+^B(OH)_2_*	12 mg/mL in 60% ACN	Automatic spraying	Adrenal gland tissue sections from pig	[79]
Dopaminergic and serotonergic neurotransmitters and their associated metabolites containing phenolic hydroxyl groups	FMP-8*, FMP-9*, FMP-10*	4.4 mM in 70% ACN	Automatic spraying, no incubation	Brain tissue sections from treated and untreated rats and primate models of Parkinsonism, brain tissue sections from a patient with Parkinson’s disease	[76]
Carbonyl as targeted functional groups					
Fluticasone propionate	DMNTH*, DNPH*	5 mg/mL in 50% ACN with 0.1% TFA, 4 mg/mL in 50% ACN	Spotting, 48 h at 37 °C	Rat lung tissue sections	[80]
11-Dehydrocorticosterone and corticosterone	GirT	0.15 mg/cm^2^, addition spraying of MeOH with 0.2% TFA	Precoated, 60 min at 40 °C	Adrenal gland tissue sections of Sprague-Dawley rat and brain tissue sections of C57BL/6 mice	[81]
Triamcinolone acetonide	GirT	5 mg/mL in MeOH with 0.2% TFA	Automatic spraying, 150 min at 40 °C	Human incubated cartilage	[82]
Testosterone	GirT	5 mg/mL in 2.5% acetic acid	Airbrush, 90 min at RT	Testis tissue sections of C57BL/6 mice after human chorionic gonadotrophin treatment	[83]
Testosterone and 5α-dihydrotestosterone	GirT	5 mg/mL in 80% MeOH with 0.1% TFA	Automatic spraying, 60 min at 40 °C	Testis tissue sections of C57BL/6 mice and prostate tissue sections of Sprague-Dawley rats after human chorionic gonadotrophin treatment	[84]
Cortisone, aldosterone, 18-oxocortisol and progesterone	GirT	10 mg/mL in 20% acetic acid	Airbrush, 90 min at RT	Adrenal gland tissue sections of human patients and Sprague Dawley rats	[85]
Abscisic acid and 12-oxo-phytodienoic acid	GirT	5 mg/mL in 80% MeOH with 2% TFA	Airbrush, 30 min at RT	Immature *P. vulgaris L.* seed sections	[86]
Pyruvic acid, glycolaldehyde, 2-pentenal, dithylacetone, 1-hexanal, 1-heptanal, jasmonic acid, dotriacontanal and more other metabolites	GirT	10 mg/mL in MeOH with 2% TFA	Electrospraying	Leaf and root tissue sections of two different maize genotypes (B73 and Mo17)	[62]
11-Dehydrocorticosterone, corticosterone	GirT	5 mg/mL in 80% MeOH with 0.2% TFA	Electrospraying, 1 h at 40 °C	Brain tissue sections of Sprague-Dawley rats	[63]
Aldosterone, cortisol, cortisone, 18-OH-corticosterone	GirT	10 mg/mL in 20% acetic acid	Airbrush, 60 min at RT	Tissue sections of human adrenal glands	[67]
Carboxylic acid as targeted functional groups					
Docosahexaenoic acid, arachidonic acid, oleic acid, palmitoleic acid, eicosapentaenoic acid, linoleic acid	PA	2 mM with 10 mM of activation reagents in ACN	Electrospraying or airbrush	Brain tissue sections from rats	[87]
3-Maleylpyruvate, N-acetyl-L-glutamate, palmitic acid, oleic acid, stearic acid and more other metabolites	PA	6 mM PA and 30 mM activation reagents in ACN	Electrospraying	Leaf and root tissue sections of two different maize genotypes (B73 and Mo17)	[62]
Fatty acids (C16:1, C16:0, C18:2, C18:1, C18:0, C18:3, C20:4, C20:0, C22:6, C22:4)	DMPI	3 mM with 1 mM HATU in 80% ACN	Electrospraying	Tumor and normal tissue sections of patients with thyroid carcinoma, brain tissue sections of rat	[88]
Thiol as targeted functional groups					
α-Chain and ß-chain of reduced insulin, glutathione, cysteine, cysteinylglycine	CHC-MAL***	10 mg/mL in 50% ACN	Automatic spraying	Liver and pancreas tissue sections of pig, tumor tissue sections of mouse xenograft	[89]
Double bond groups					
PC 36:1, PS 36:2 isomers	Benzaldehyde	Vapor of benzaldehyde	Custom-made reaction chamber, triggered by 254 nm	Brain tissue sections of C57BL6/N mice	[90]
PC 34:1, PC 36:1 isomers	BPh*	20 mg/mL in solution (ACN: isopropanol: H_2_O, 6:3:1 with 0.5% TFA)	3 min with UV-light	Brain tissue sections of C57BL6/N mice and tegument of *S. mansoni*	[91]
PC 34:1, PC 36:1 isomers	Ozone	Gas	High-pressure linear ion trap with ozone	Brain tissue sections of ND2:SmoA1 transgenic mice containing tumors	[92]
PC 34:1 isomers	Ozone	Ozone from an ozone generator	Glass flask flushed with the flow of ozone, up to 30 min	Brain tissue sections of BALB/c mice, human colon tissue sections	[93]

*CA*, 4-hydroxy-3-methoxycinnamaldehyde; *NBA*, 2-nitrobenzaldehyde; *TAHS*, p-*N*,*N*,*N*-trimethylammonioanilyl *N*-hydroxysuccinimidyl carbamate iodide; *DPP-TFB*, 2,4-diphenyl-pyranylium tetrafluoroborate; *DPP*, 2,4-diphenyl-pyranylium; *PBDPP*, 1,4-phenylene-4,4′-bis (2,6-diphenyl-4-pyrylium); *TMP*, 2,4,6-trimethylpyrylium; FMP-8: 4-(10-bromoanthracen-9-yl)-2-fluoro-1-methylpyridin-1-ium iodide; *FMP-9*, 4-(anthracen-9-yl)-2-fluoro-1-ethylpyridin-1-ium iodide; *FMP-10*, 4-(anthracen-9-yl)-2-fluoro-1-methylpyridin-1-ium iodide; *FMPTS*, 2-fluoro-1-methylpyridinium *p*-toluenesulfonate; *(N-Me)Py+B(OH)*_*2*_, 4-(N-methyl)pyridinium boronic acid; *DNPH*, 2,4-dinitrophenylhydrazine; *DMNTH*, 4-dimethylamino-6-(4-methoxy-1-naphthyl)-1,3,5-triazine-2-hydrazine; *GirT*, Girard’s reagent T; *PA*, 2-picolylamine (PA); *DMPI*, N,N-dimethylpiperazine iodide; *CHC-Mal*, (*E*)-2-cyano-N-(2-(2,5-dioxo-2,5-dihydro-1H-pyrrol-1-yl)ethyl)-3-(4-hydroxyphenyl)-acrylamide (CHC-Mal); *BPh*, benzophenone; *ACN*, acetonitrile; *TFA*, trifluoroacetic acid; *EtOH*, ethanol; *FA*, formic acid; *TEA*, triethylamine; *RT*, room temperature; *HATU*, 2-(7-azabenzotriazol-1-yl)-N,N,N´,N´-tetramethyluronium hexafluorophosphate; *MeOH*, methanol

*Reactive matrix; **Reactive matrix in high concentration; ***Reactive matrix for small thiol-containing molecules

### Target functional group: Amine

#### With *trans*-cinnamaldehyde/4-hydroxy-3-methoxycinnamaldehyde

The functional aldehyde group reacts easily with primary amines, forming a stable Schiff’s base. Using this reaction, Manier and coworkers [59] investigated *trans*-cinnamaldehyde for the derivatization of isoniazid, an anti-tuberculosis drug, in rabbit lung tissue sections. Later, Manier and coworkers [60] applied 4-hydroxy-3-methoxycinnamaldehyde (CA) for the visualization of dopamine, norepinephrine, and epinephrine in porcine adrenal gland tissue sections and γ-aminobutyric acid in rat brain tissue sections. The pre-coating method of the CA resulted in enhanced sensitivity with minimal analyte delocalization. CA was also used by Esteve and coworkers [61] for the comparison with THAS and DPP-TFB. Dueñas and coworkers [62] also applied CA for a proof of concept experiment, in which various classes of compounds containing a primary amine group were visualized in tissue sections of maize leaves and roots. Guo and coworkers [63] combined a laser-assisted tissue transfer (LATT) technique with the CA derivatization for imaging of up to 67 small molecule metabolites including amino acids, neurotransmitters and dipeptides, and others in brain tissue sections of rats.

#### With NBA and the nanosecond laser irradiation

Li and coworkers [64] introduced the photoactive compound, 2-nitrobenzaldehyde (NBA) in combination with the nanosecond laser irradiation at a wavelength of 355 nm for the derivatization of primary amine groups in peptides and proteins. The nanosecond laser irradiation enables the generation of the reactive 2-nitrosobenzoic anion (NS^−^) which can rapidly react with primary amines in peptides and proteins, resulting in a mass shift of 133 Da. It was called as a nanosecond photochemical reaction (nsPCR). Base on the established microelectrophoresis and thermophoresis theory, they also proposed the on-demand matrix-removal effect for the NBA-based nsPCR strategy. The NBA-based nsPCR strategy could be applied to the brain tissue sections of the mouse brain to visualize and identify neuropeptides in the mouse brain with massive enhanced results.

#### With TAHS

Originally, p-N,N,N-trimethylammonioanilyl N-hydroxysuccinimidyl carbamate iodide (TAHS) was synthesized by Shimbo and coworkers [94] using N,N-dimethylamino-p-phenylenediamine and N,N′-dihydroxysuccinimidyl carbonate and iodomethane for the subsequent methylation and applied it for the analysis of amino acids in ESI-MS. According to this synthesis method, Toue and coworker [65] synthesized THAS for derivatization of amino acids in liver tissue sections from xenograft mouse models of human colon cancer in positive ion mode. After the derivatization with TAHS and subsequent application of 2,5-DHB matrix, the detection sensitivity of amino acids could be improved from failure in detection to easily detection. Most amino acids could be easily detected in tissue sections, especially because of the introduction of the positively charged quaternary amine group. However, some amino acids, such as lysine, histidine, threonine, aspartate, and arginine [61, 94], could hardly be detected. Further application of TAHS could be found for the analysis of phenylalanine and tyrosine [66] in liver tissue sections from a H460 human NSCLC xenograft mouse model, noradrenaline [67] in adrenal gland tissue sections from tumor patients, and glutamine [68] in tumor and benign tissue sections from patients with cholangiocarcinoma.

#### With DPP-TFB

Conversion of the primary amino groups into charged quaternary amino groups is a good option to increase ionization efficacy. Shariatgorji and coworkers [69, 70] applied the commercially available pyrylium salts for the derivatization of primary amino groups in neurotransmitters and amino acids. They selected pyrylium salt such as 2,4-diphenyl-pyranylium tetrafluoroborate (DPP-TFB), which reacts selectively and rapidly with primary amine groups under mild conditions. The resulting charged derivatization product possessed the ability of self-assisted laser desorption ionization in positive ion mode, but only with a higher concentration of DPP-TFB solution and in the presence of high amounts of metabolites. Therefore, an additional matrix such as CHCA [69] or 2,5-DHB [71–75] was applied onto brain tissue sections after derivatization, leading to the enhanced sensitivity in MALDI-MSI analysis. DPP-TFB [69–75] was applied in various animal models to localize and (semi-)quantitatively estimate changes of the metabolites such as dopamine, tyrosine, tryptamine, tyramine, phenethylamine, 3-methoxytyramine, serotonin, γ-aminobutyric acid, glutamate, glycine, alanine, proline, valine, threonine, taurine, leucine, aspirate, glutamine, lysine, L-dihydroxyphenylalanine, histamine, methylhistamine, agmatine, adenine, and norepinephrine.

#### With FMP reagents

Shariatgorji and coworkers [76] synthesized a series of pyridinium salts including 4-(10-bromoanthracen-9-yl)-2-fluoro-1-methylpyridin-1-ium iodide (FMP-8), 4-(anthracen-9-yl)-2-fluoro-1-ethylpyridin-1-ium iodide (FMP-9) and 4-(anthracen-9-yl)-2-fluoro-1-methylpyridin-1-ium iodide (FMP-10), which readily reacted with primary or secondary amine groups and were proven as the best candidates among them. The derivatization reaction follows the nucleophilic aromatic substitution reaction of the 2-fluoro-1-methyl pyridinium (FMP) cation with primary and secondary amine groups, which are often the functional groups of neurotransmitters and their associated metabolites from brain tissue sections. Simultaneously, the polyphenyl group in FMP reagents complies with the requirement of a MALDI matrix, which has a strong light absorption in the range of ultraviolet-visible in the solid phase. Hence, the chemically derivatized brain tissue sections can be directly measured with MALDI mass spectrometer without spraying of an additional matrix. FMP reagents were dubbed as reactive matrices. The improved sensitivity after derivatization with FMP reagents enabled a comprehensive map of neurotransmitters and their associated metabolites from brain tissue sections. The ability to form small FMP derivative crystals enabled the application at a high spatial resolution of 10 μm. Zhang and coworkers [77] from the same laboratory applied FMP-10 to visualize the dopamine and γ-aminobutyric acid changes in parkinsonian mice lacking GPR37 in comparison with wild type mice.

### Target functional group: phenolic hydroxyl

#### With FMP

Beasley and coworkers [78] applied 2-fluoro-1-methylpyridinium *p*-toluene sulfonate (FMPTS) for derivatization of the phenolic hydroxyl group of cannabinoids in human hair. After rapid derivatization reaction at room temperature and spraying of CHCA as the MALDI matrix, positively charged N-methylpyridinium derivate contributed to improved ionization efficiency of several cannabinoids and their metabolites including Δ^9^-tetrahydrocannabinol (THC) in hair, which were soaked in solutions with the respective analytes. Furthermore, THC could be visualized with FMPTS derivatization in hairs from a known cannabis consumer.

#### Derivatization of diol in catechol with (N-Me)Py^+^B(OH)_2_

Kaya and coworkers [79] synthesized 4-(N-methyl)pyridinium boronic acid ((N-Me)Py^+^B(OH)_2_), which was applied for derivatization of catecholamines including dopamine, epinephrine, and norepinephrine in porcine adrenal gland tissue sections. Boronic acid derivative can readily react with the diol moiety of catechol (*ortho* isomer of dihydroxybenzene), forming five-membered boronate esters. Due to the positive charge in (N-Me)Py^+^B(OH)_2_ and the UV-absorption ability of the derivatization products in the range of the laser wavelength of the mass spectrometer, (N-Me)Py^+^B(OH)_2_ was used as a reactive matrix without an additional MALDI matrix. Additionally, the unique isotopic pattern of boron-containing derivatized catecholamines assisted in data analysis.

### Target functional group: carbonyl

#### With Girard’s reagent T

Cobice and coworkers [81] applied Girard’s reagent T (GirT) for derivatization of 11-dehydrocorticosterone and corticosterone in rat adrenal gland and mouse brain tissue sections. The reactive hydrazine group in GirT reacts with the ketone group easily, forming hydrazine derivatives. Due to the charged quaternary amine in GirT, the resulting derivatization products (GirT-dehydrocorticosterone and GirT-corticosterone) showed enhanced ionization efficiency. Derivatization with GirT to visualize ketone-containing analytes was demonstrated in various tissues, such as mouse testis [83, 84], cartilage [82], rat prostate [84], rat adrenal gland [85], immature *P. vulgaris L.* seed [86], maize root and leaf [62], human adrenal gland [67, 85], and rat brain [63]. In the case of structural isomers [67], tandem MS imaging was used to distinguish aldosterone from cortisol and cortisone from 18-OH-corticosterone.

#### DMNTH and DNPH

Flinder and coworkers [80] applied different hydrazines including 2,4-dinitrophenylhydrazine (DNPH) and 4-dimethylamino-6-(4-methoxy-1-naphthyl)-1,3,5-triazine-2-hydrazine (DMNTH) on mouse lung tissue sections, which were spotted with fluticasone propionate. DNPH and DMNTH were demonstrated as reactive matrix and can be obtained by purchase and chemical synthesis in the lab, respectively. In comparison with DNPH, DMNTH showed superior results and resulted in a detection limit of 50 ng/μL in a humid environment at 37 °C for 48 h. The detection sensitivity was improved after additional application of conventional MALDI matrix CHCA.

### Target functional group: carboxylic acid

#### With 2-picolylamine

Wu and coworkers [87] evaluated 2-picolylamine (PA), well known as derivatization reagent in solution for application as OTCD before MALDI-MSI. After activating of a carboxylic acid with 2,2-dipyridyl disulfide (DPDS) and triphenylphosphine (TPP), 2-picolylamine transferred the activated fatty acid to the stable derivatization product containing an amide bond. By using the electrospray deposition method, six fatty acids, such as docosahexaenoic acid, arachidonic acid, oleic acid, palmitoleic acid, eicosapentaenoic acid, and linoleic acid, were visualized in rat brain tissue sections with improved detection limits and decreased delocalization in positive ion mode. Thus, a spatial resolution of 20 μm was reached. The approach was also applied by Dueñas and coworkers [62] for visualization of fatty acids, such as 3-maleylpyruvate, N-acetyl-L-glutamate, palmitic acid, oleic acid, stearic acid, and more other metabolites with carboxylic acid in tissue sections of maize leaves and roots.

#### With DMPI

Wang and coworkers [88] synthesized N,N-dimethylpiperazine iodide (DMPI) using CH_3_I and N-methylpiperazine. 2-(7-Azabenzotriazol-1-yl)-N,N,N´,N´-tetramethyluronium hexafluorophosphate (HATU) was used in this work to generate active esters from carboxylic acids derived from fatty acids such as C16:0 and C18:0. The active esters reacted then with DMPI, resulting in derivatized fatty acids with stable amide bonds and charged quaternary amine groups, which assisted in improved detection of fatty acid derivatives in positive ion mode. In this way, fatty acids and phospholipids could be simultaneously imaged in positive ion mode. By using optimized spraying parameters using an electrospray device and optimized derivatization conditions, this approach was proved to have advantage over the approach with 2-picolyamine [87] and applied in tissue sections from patients with thyroid cancer.

### Target functional group: thiol

Fülöp and coworkers [89] designed, synthesized, and evaluated (E)-2-cyano-N-(2-(2,5-dioxo-2,5-dihydro-1H-pyrrol-1-yl)ethyl)-3-(4-hydroxyphenyl)-acrylamide (CHC-Mal) as a thiol derivatization reagent. Proteins with free thiol groups, such as α-chain and ß-chain of reduced insulin, were derivatized and imaged in porcine pancreas tissue sections in positive ion mode. Besides, free thiol groups from small molecule metabolites, present in tissue or generated by spray-coated reducing agent, were derivatized and visualized in porcine liver tissue sections and tumor tissue sections of a mouse xenograft model in positive ion mode with clearly improved detection sensitivity. In this work, cysteine, cysteinylglycine, and glutathione were detectable even without application of additional 2,5-DHB matrix. In other words, CHC-Mal can be used as a reactive matrix for small thiol-containing metabolites.

### Target functional group: double bond

#### With benzaldehyde/BPh

Bednařík and coworkers [90] introduced benzaldehyde for distinguishing and imaging of lipid double bonds. A laser-induced post-ionization strategy (MALDI-2) was used for improved protonation of analytes. Benzaldehyde undergoes the Paternò-Büchi photoreaction with lipid double bonds in a reaction chamber equipped with a UV lamp. After derivatization and subsequent collision-induced dissociation of the Paternò-Büchi photoreaction reaction products, the double bond position were verified and imaged in mouse brain tissue. Wäldchen and coworkers [91] introduced benzophenone (BPh) as a reactive matrix for the identification of the double bond position of unsaturated phospholipids. BPh could not only act as a MALDI matrix but also react with double bond groups of unsaturated phospholipids directly after laser irradiation (330–370 nm) during measurement. Thus, no additional equipment is required for the laser-induced Paternò-Büchi photoreaction, in which C=C double bond form a four-membered oxetane ring. Using tandem MS imaging (MS^2^I), spatial distributions of phospholipids with defined double bond position could be visualized in brain tissue sections of mice and tegument of *S. mansoni*.

#### With ozone

Paine and coworkers [92] directly introduced ozone in the mass spectrometer, leading to ozone-induced dissociation. With this unique approach, specific lipid isomers were visualized in the rat brain. Later, Bednařík and coworkers [93] developed the approach for ozonization of unsaturated lipids under concentrated ozone atmosphere in a reaction chamber. The Criegee ozonide ions are formed after the addition of ozone to C=C double bond in PC 34:1 and the subsequent rearrangement. Higher-collisional energy dissociation (HCD) of Criegee ozonide ions results in an aldehyde and carboxylic acid fragments which can indicate the C=C double bond position. Δ9 and Δ11 isomers of PC 34:1 in brain tissue sections of BALB/c mice and human colon tissue sections could be visualized using this technique by MS^2^I.

## Conclusion

In this review, we have summarized recent advances in the design, discovery, and characterization of novel small organic matrices and OTCD reagents to improve MALDI-MSI performance. Although many novel matrices are still not designed for broader application, MALDI-MSI with novel matrices demonstrated to have superior results than conventional matrices in many aspects, for example, vacuum stability, detection sensitivity, molecular coverage, salt tolerance, matrix background signals, ion suppression, and crystal formation. Novel matrices have the potential to improve image quality and expand the application area of MALDI-MSI. Nevertheless, the possibility of theoretical simulations for matrix design is still missing for ionization efficiency, interaction of matrices with other competent metabolites in tissue sections, crystal formation, and other matrix performances. At the same time, OTCD processes for MALDI-MSI were proven to be promising methods for visualization of poorly ionizable analytes, distinction of isomers, and characterization of isomers. Accompanying problems found in OTCD processes, such as low reaction efficiency, poor reproducibility, and additional time consumption, constrain attempts to transfer and optimize versatile in-solution chemical derivatization for OTCD for MALDI-MSI. However, OTCD processes in MALDI-MSI workflow have clear advantages in the specific detection of analytes in tissue sections. Thus, more and more attention will be paid for transferring and optimizing chemical derivatization in tissue sections, opening new possibilities for MALDI-MSI to become an indispensable tool in different research fields.
